# Circular ZDHHC11 supports Burkitt lymphoma growth independent of its miR-150 binding capacity

**DOI:** 10.1038/s41598-024-59443-3

**Published:** 2024-04-16

**Authors:** Yichen Liu, Xing Zhao, Annika Seitz, Annie A. Hooijsma, Reyhaneh Ravanbakhsh, Sofia Sheveleva, Debora de Jong, Jasper Koerts, Agnieszka Dzikiewicz-Krawczyk, Anke van den Berg, Lotteke J. Y. M. Ziel-Swier, Joost Kluiver

**Affiliations:** 1 Department of Pathology and Medical Biology, University of Groningen, University Medical Center Groningen, Hanzeplein 1, 9700 RB Groningen, The Netherlands; 2 Cancer Hospital Academy of Medical Sciences, Peking Union Medical College, Dongcheng, China; 3 Department of Aquatic Biotechnology, Artemia and Aquaculture Research Institute, Urmia University, Urmia, Iran; 4 Institute of Human Genetics, Polish Academy of Sciences, Poznan, Poland

**Keywords:** Lymphoma, Oncogenes, Long non-coding RNAs, miRNAs

## Abstract

We previously showed that MYC promoted Burkitt lymphoma (BL) growth by inhibiting the tumor suppressor miR-150, resulting in release of miR-150 targets MYB and ZDHHC11. The *ZDHHC11* gene encodes three different transcripts including a mRNA (pcZDHHC11), a linear long non-coding RNA (lncZDHHC11) and a circular RNA (circZDHHC11). All transcripts contain the same region with 18 miR-150 binding sites. Here we studied the relevance of circZDHHC11, including this miR-150 binding site region, for growth of BL cells. CircZDHHC11 was mainly present in the cytoplasmic fraction in BL cells and its localization was not altered upon miR-150 overexpression. Knockdown of circZDHHC11 caused a strong inhibition of BL growth without affecting the expression levels of MYC, MYB, miR-150 and other genes. Overexpression of circZDHHC11 neither affected cell growth, nor rescued the phenotype induced by miR-150 overexpression. Genomic deletion of the miR-150 binding site region did not affect growth, nor did it change the effect of circZDHHC11 knockdown. This indicated that the miR-150 binding site region is dispensable for the growth promoting role of circZDHHC11. To conclude, our results show that circZDHHC11 is a crucial factor supporting BL cell growth independent of its ability to sponge miR-150.

## Introduction

In our previous work, we have shown expression of a circular RNA (circZDHHC11) derived from the *ZDHHC11* locus in BL^1^. This gene also encodes a protein-coding (pcZDHHC11) and a long non-coding RNA (lncRNA, lncZDHHC11). Knockdown of ZDHHC11 using shRNAs targeting all three transcripts simultaneously resulted in a strong decrease in BL cell growth.

Circular RNAs (circRNAs) are formed through a process called back-splicing of RNA transcripts. The circular shape is generated by a covalent link between the 5’ and 3’ ends of an RNA molecule. The region where these ends are linked is known as the back-splice junction (BSJ) region^2^. CircRNAs are more stable than linear RNAs because they do not contain free 5’ and 3’ ends, which makes them resistant to degradation by exonucleases^3^. Thousands of circRNAs have been identified in eukaryotes through RNA sequencing and bioinformatics analyses, which have focused on the identification of reads containing BSJ sequences. About 85% of human circRNAs align to known genes, with the majority being derived from protein-coding exons and a smaller proportion originating from untranslated regions (UTRs) or introns. In addition, circRNAs can be also derived from intergenic regions or from antisense transcripts of known genes^4^. CircRNAs can encompass single or multiple exons, with or without the presence of (part of) introns. The size of circRNAs ranges from 100 nt to more than 4 kb^5^.

CircRNA profiling showed cell and tissue type specific expression patterns^6,7^. For a substantial subset of circRNAs, this is likely due to the fact that they follow the expression of their host gene. However, for a smaller subset of circRNAs, expression patterns were not associated with the expression patterns of their host genes^8^. Most circRNAs are located in the cytoplasm, while a smaller subset of the circRNAs arepresent in the nucleus^4^. Characterization of circRNAs has revealed a broad spectrum of different functionalities. Some circRNAs can serve as sponges of microRNAs and thereby compete with other RNA transcripts for binding to microRNAs^9^. However, as many circRNAs are expressed at relative low levels and contain a limited number of miRNA binding sites, it is thought that only a minority of circRNAs can function as effective miRNA sponges^10^. Besides binding to microRNAs, circRNAs can also interact with DNA, mRNA and proteins, thereby affecting transcription^11^, RNA splicing^12^, and mRNA stability^13,14^ and translation. In addition, it has also been shown that circRNAs themselves can be translated into proteins and give rise to novel proteins by including the unique BSJ region in the open reading frame^13,15–17^. Based on all these potential interactions, it has become clear that the functionality of circRNAs is quite diverse^18^.

CircRNAs have been implicated in human diseases including different types of cancers^19,20^. Some circRNAs show oncogenic properties^21–23^, while others show tumor suppressor activity^24–26^. Relatively little is known about the role of circRNAs in B-cell lymphoma. Initial studies have characterized circRNA expression and/or function in Burkitt lymphoma (BL), diffuse large B-cell lymphoma (DLBCL), mantle cell lymphoma (MCL) and follicular lymphoma (FL)^1,27–31^.

The ZDHHC11 gene contains 18 miR-150 binding sites, which are present in all three transcripts. This high number of binding sites is quite unique and supports a potential role as miRNA sponge. The postulated functional mechanism of the ZDHHC11 transcripts in regulating growth of BL was to ensure high levels of MYB by binding to miR-150^1^. Enrichment of circZDHHC11 in the AGO2 immunoprecipitated fraction upon miR-150 overexpression was more pronounced than enrichment of pcZDHHC11 or lncZDHHC11 indicating a strong interaction with miR-150^1^. Based on these findings, we hypothesized that circZDHHC11 might play a dominant role in regulating growth of BL by sponging the available miR-150. In this study, we investigated the relevance of circZDHHC11 and the miR-150 binding site region in regulating growth of BL. We studied the effects of knockdown and overexpression of circZDHHC11 in unmodified and in miR-150 binding site knockout BL cells.

## Materials and methods

### Cell lines and culturing

Burkitt lymphoma cell lines ST486, BL41, CA46 and DG75 and the HEK293T cell line were purchased from ATCC and DSMZ. ST486 was cultured in RPMI 1640 (Thermo Fisher, Waltham, MA, USA) containing 20% fetal bovine serum (Sigma, St. Louis, MO, USA). DG75, CA46, and BL41 were cultured in RPMI 1640 (Thermo Fisher) containing 10% fetal bovine serum. HEK293T was cultured in DMEM (Thermo Fisher) containing 10% fetal bovine serum. All media were supplemented with 2 mM glutamine, 100 U/mL of penicillin and 100 µg/mL of streptomycin (Thermo Fisher). Cells were cultured at 37 °C in a humidified air atmosphere supplemented with 5% CO_2_. The origin of the cell lines was confirmed with STR DNA analyses on a regular basis and mycoplasma tests were performed to exclude contamination.

### Lentiviral constructs and viral transfections

To knockdown expression of circZDHHC11, shRNAs against the BSJ region (Supplemental Fig. 1) were designed and cloned into a miRZip-GFP lentiviral vector. Non-targeting shRNAs were used as controls. To overexpress miR-150, we used a pCDH lentiviral vector containing the miR-150 stem-loop and flanking regions. As a control we included the pCDH vector without an insert (EV). Vectors were all purchased from System Biosciences (Palo Alto, CA, USA). To overexpress circZDHHC11, a PLC5-circ vector-based construct containing the full-length circZDHHC11 was purchased from Geneseed (Guangzhou, China). To delete the miR-150 binding site region we generated a construct which expresses two sgRNA sequences (Supplemental Table 1). This construct was made by inserting a minigene containing both sgRNA sequences as well as the gRNA scaffold for the first sgRNA and the H1 promoter sequence upstream the second sgRNA into the lentiCRISPR-EV-GFP vector (Addgene, Watertown, MA, USA). Lentiviral particles were generated in HEK293T cells as published previously and were either used directly to infect target cells or stored at -80°C after being harvested^32^.

**Figure 1:**
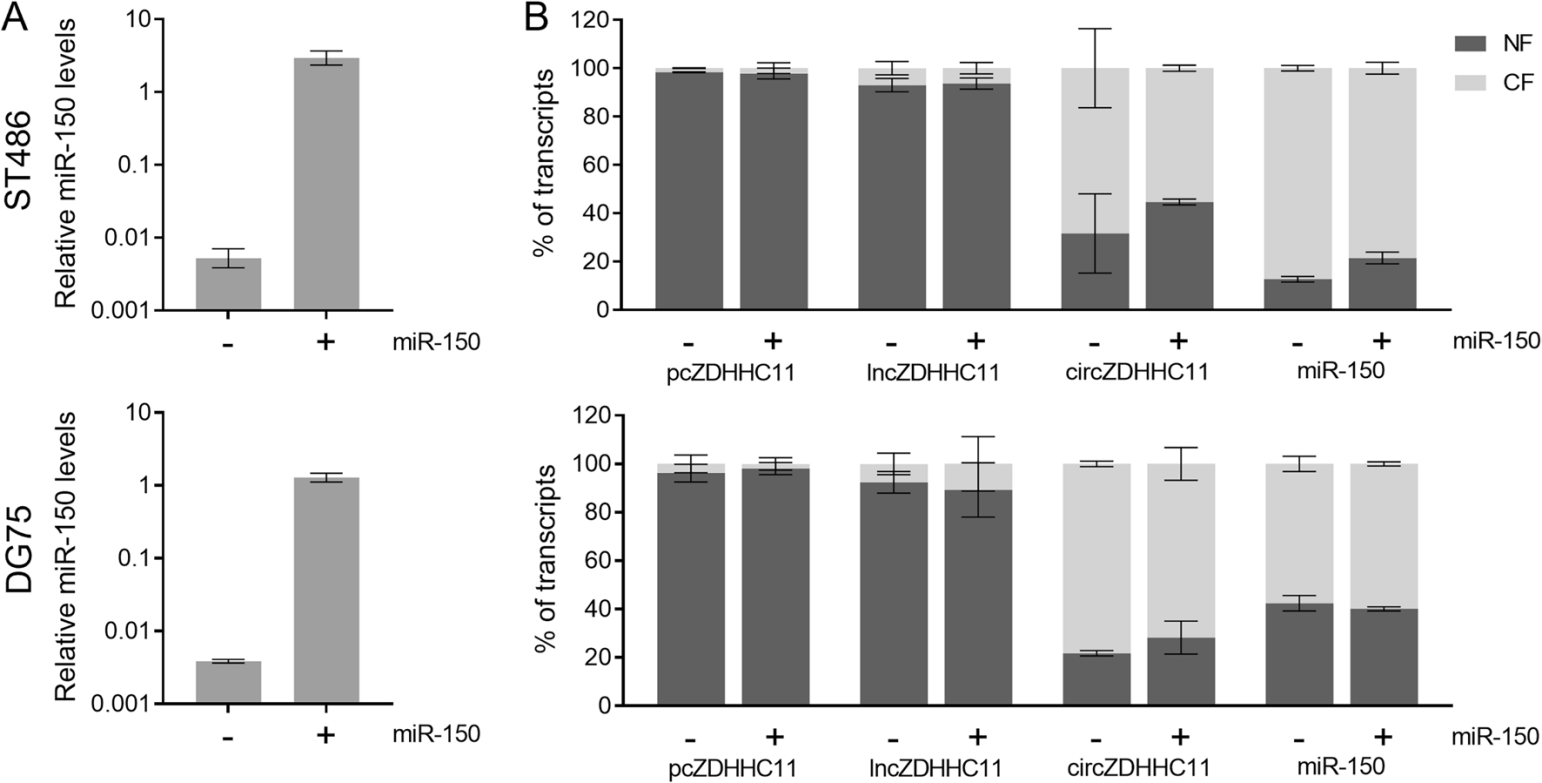
Subcellular localization of the *ZDHHC11* transcripts and miR-150 in BL cells with and without overexpression of miR-150. (**A**) Exogenous expression levels of miR-150 in ST486 and DG75 upon infection with a lentiviral control vector (-) or miR-150 overexpression vector ( +). (**B**) Subcellular localization of the *ZDHHC11* transcripts and miR-150 in the absence (-) or presence ( +) of miR-150 overexpression in ST486 and DG75 cells. The fraction of the total RNA present in the cytoplasm (light grey, CF) and nucleus (dark grey, NF) is indicated. Error bars indicate the mean ± SD of two independent experiments.

### Generation of monoclonal miR-150 binding site depleted ST486 cell line

ST486 cells were infected with a lentiviral vector containing two single guide RNAs flanking the miR-150 binding site region and the Cas9 sequence to remove the miR-150 binding site region of the ZDHHC11 locus. As a control, a lentiviral vector with two non-targeting sgRNAs was used. Both vectors contain a GFP reporter, allowing sorting of GFP^+^ cells using a MoFlo sorter (BD Biosciences, Franklin Lakes, NJ, USA). Cells were plated into 96 wells plates containing 100 µL of 1:8 normal medium:Methocult medium (Stemcell Technologies, Vancouver, Canada), aiming at 1 cell per well. Cultures were subsequently expanded to obtain sufficient cells for isolation of genomic DNA, which was performed with a standard salt-chloroform protocol. To characterize the deletion , 100 ng of DNA was used as template in a PCR reaction with 0.033 U/µL of Taq polymerase (Invitrogen, Waltham, MA, USA) and 0.5 µM of primers in 30 µl reaction volume. After purification using the DNA Clean & Concentrator kit (Zymo Research, Irvine, CA, USA), the PCR product was sequenced by Eurofins Genomics (Ebersberg, Germany) using primers flanking the binding site region. Primer sequences are shown in Supplemental Table 1.

### Growth competition assays

Cells were infected with a lentiviral vector carrying a shRNA targeting circZDHHC11 or a transgene (for overexpression of miR-150 or circZDHHC11) together with a reporter gene. We aimed at infection percentages of 10–50%. Cells infected with lentiviral vectors containing non-targeting or scrambled sequences were used as controls. Vectors contained GFP, RFP or BFP, and these reporters were used to follow the percentages of infected cells by flow cytometry over time on a BD Accuri C6 Plus Cell Analyzer (BD Biosciences), Calibur flow cytometer (BD Biosciences) or NovoCyte Quanteon flow cytometer (Agilent Technologies, Santa Clara, CA, USA). Percentages of infected cells were measured at day 4 post-transfection and monitored tri-weekly for three weeks. Data were analyzed using the FlowJo software (version 10, Treestar, BD Biosciences). To determine the effect on cell growth, the percentage of GFP/RFP/BFP positive cells at day 4 was set to 100% and the fold difference relative to this starting point was calculated for each time point. The mixed model analysis was performed as previously described to compare the difference of relative GFP/RFP/BFP percentages at day 22 post-transfection^1^.

### Cell fractionation

Cell pellets were washed in 10 mM Tris, pH 8.0, 10 mM NaCl, 2 mM MgAc_2_, 300 mM sucrose, 0.5 mM DTT, 4 U/mL RNase inhibitor (Ambion/Life Technologies, Carlsbad, CA, USA) and EDTA-free protease inhibitor cocktail (Roche, Basel, Switzerland). Subsequent cell lysis was performed in the same buffer supplemented with 3 mM CaCl2 and 0.1% NP-40. After pelleting the nuclear fraction at 1000 × g for 5 min at 4°C, the supernatant was collected as the cytoplasmic fraction. The nuclear fraction was resuspended in 50 mM Tris, pH 8.0, 5 mM MgAc_2_, 25% glycerol, 0.1 mM EDTA, 5 mM DDT, 20 U/mL RNase inhibitor and EDTA-free protease inhibitor cocktail, and harvested by centrifugation at 1000 × g for 5 min. The fractions were resuspended in Qiazol buffer for analysis by RT-qPCR or in Cell lysis buffer for analysis by Western blot.

### RNA isolation, RT-qPCR and PCR

Cells or cell fractions were resuspended in 700 µl of Qiazol lysis buffer, and RNA was purified using the miRNeasy micro or mini kit (Qiagen, Hilden, Germany) together with Phase Lock Gel Heavy tubes (5 Prime) according to the manufacturer’s protocol. The synthesis and amplification of cDNA were performed as described previously, using 500 ng of RNA^1^. For the subcellular localization experiments, equal volumes of the nuclear fraction and cytoplasm fraction were used for cDNA synthesis. For quantification of the circZDHHC11 levels, 5–10 ng of cDNA was used as input in 10 µL Sybr-Green (Applied Biosystems, Waltham, MA, USA) qPCR reactions, while 1 ng was used for the other genes. For the quantification of miRNA levels, multiplex miRNA-specific cDNA synthesis and RT-qPCR were performed using the Taqman MicroRNA Reverse Transcription Kit and Taqman MicroRNA Assays (Applied Biosystems) according to the manufacturer’s protocol. For validation of the exogenous circZDHHC11 sequence, PCRs were performed with 0.033 U/µL of Taq polymerase (Invitrogen) and an input of 10 ng of cDNA and 0.5 µM of primers in 30 µl reaction volume. After purification of the PCR products using the DNA Clean & Concentrator kit (Zymo Research), they were sequenced by Eurofins Genomics. All primers used are listed in Supplemental Table 1.

### Western blot

Cells or cell fractions were resuspended in Cell lysis buffer (Cell Signaling, Danvers, MA, USA) supplemented with 1 mM PMSF. After incubation on ice for 45 min and centrifugation for 10 min at 14,000 rpm and 4°C, the lysate was collected, and the total protein concentration was determined using the Pierce BCA Protein Assay Kit (ThermoFisher). SDS-PAGE was performed using 10% or 12% polyacrylamide gels and subsequent transfer onto nitrocellulose membranes was done as described previously^32^. Membranes were blocked in 5% ELK in TBST, followed by incubation with antibodies diluted in 5% ELK in TBST. The antibodies used are listed in Supplemental Table 2. Membranes were incubated with SuperSignal West Pico Chemiluminescent Substrate (ThermoFisher). The proteins of interest were visualized using a ChemiDoc MP scanner and quantified using the Image Lab 6.0 software (BioRad, Hercules, CA, USA). Protein levels were normalized to GAPDH. For stripping of the membranes, incubation in stripping buffer (25 mM glycine, pH 2.0, 0.001% SDS) for 15 min was followed by blocking in 5% ELK in TBST for 15 min, after which incubation with antibodies was performed.

### Microarray

ST486 cells were infected with shRNAs (sh1 and sh2) against circZDHHC11 or control shRNA (NT1). Two independent experiments were performed and based on the steepest decline in GFP competition assay, GFP + cells were sorted (> 98% purity) on day 5 (exp 1) or day 6 (exp 2) post-infection. As a quality control unsorted cells from the same infection were followed in a GFP competition assay to confirm the expected phenotype and qRT-PCR was performed to confirm efficient knockdown of circZDHHC11. RNA was isolated as described above and labeled with Cyanine 3-CTP using the LowInput QuickAmp Labeling kit and Cyanine 3 CTP Dye Pack (both Agilent Technologies) according to the manufacturer’s protocol. Cy3-labeled cDNA samples were hybridized on an Agilent SurePrint G3 Human GE 8 × 60 microarray (Agilent ID 72363) slide overnight. Resulting raw data were analyzed with Gene-Spring GX 12.5 software (Agilent Technologies) using quantile normalization without baseline transformation. Probes used for further analyses were flagged as present by the feature extracting software in all conditions resulting in 20,838 usable probes out of a total of 58,341 probes. The raw and normalized microarray data were deposited in the Gene Expression Omnibus database (http://www.ncbi.nlm.nih.gov/geo; accession number GSE252484). Statistically significant changes between the shRNAs (sh1 + sh2) compared to the control shRNA were determined by a moderated t-test using Benjamini–Hochberg multiple testing correction combining both experiments.

### Confirmation of circular RNA structure by RNase R treatment

Total RNA (1 μg for each sample) was incubated for 15 min at 37 °C with 1 U Rnase R (Biosearch Technologies, Hoddesdon, UK) in a final reaction volume of 15 μL. RNA samples without RNase R were included as controls. After incubation, the RNA was concentrated using a Vivacon® 500 Hydrosart filter (Sartorius, Göttingen, Germany). RNA was replenished to the same volume with H_2_O and RT-qPCR was performed to test the abundance of linear and circular RNA using the same volume of RNA as described above.

### AGO2 RNA immunoprecipitation

Immunoprecipitation of AGO2-containing RISC complexes was performed as described previously with minor modifications^33^. EZview protein G beads (Merck Life Science NV, Darmstadt, Germany) were pre-blocked using 5% BSA and 2 µg/µL of salmon sperm sonicated ssDNA (Merck Life Science NV), before they were incubated with 10 µg/mL anti-AGO2 antibody (clone 2E12-1C9, Abnova). Mouse anti-IgG antibody was used as negative control. Following the immunoprecipitation procedure, RNA from the total, flow through and immunoprecipitation fractions were isolated and analyzed by qPCR as described above.

## Results

### Subcellular localization of circZDHHC11

Since the function of circRNAs is related to their subcellular localization, we studied the abundance of circZHHC11 in the nucleus and cytosol (Fig. 1 and Supplemental Figs. 2 and 3). We analyzed BL cells with and without miR-150 overexpression to determine whether miR-150 overexpression has an effect on the subcellular localization of the three ZDHHC11 transcripts. Infection of BL cells with lentiviral vectors containing the miR-150 precursor sequence revealed a prominent induction of miR-150 levels in both BL cell lines (Fig. 1A). Efficient fractionation was validated by WB and RT-qPCR (Supplemental Fig. 2 and 3). The pcZDHHC11 and lncZDHHC11 transcripts showed a predominantly nuclear localization with more than 90% of the transcripts residing in the nucleus (Fig. 1B). In line with our previous observations showing that circZDHHC11 and miR-150 strongly interact, we observed that both RNA molecules were located predominantly in the cytosol. We did not detect any obvious change in subcellular localization of the individual ZDHHC11 transcripts upon miR-150 overexpression.

**Figure 2:**
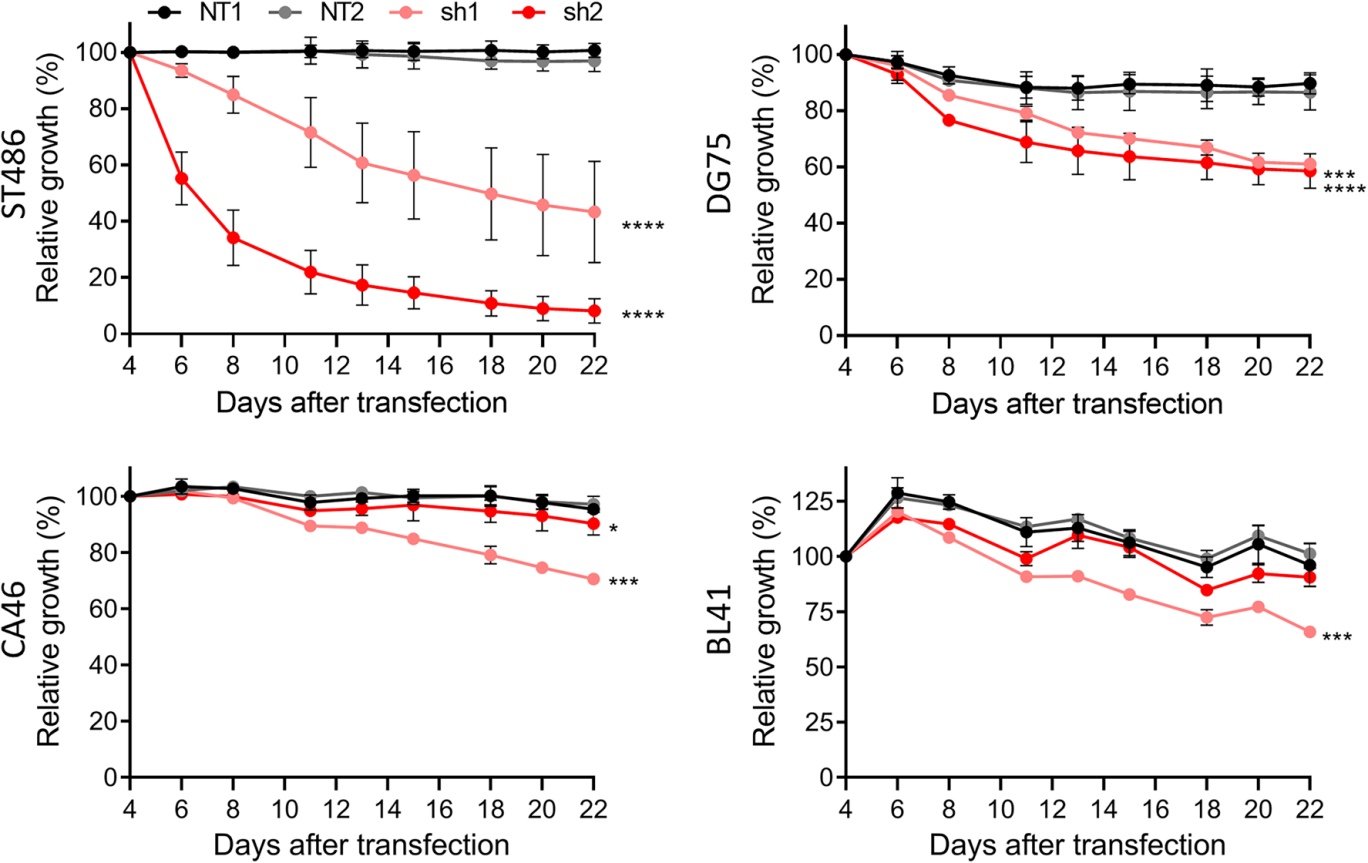
CircZDHHC11 knockdown inhibits Burkitt lymphoma cell growth. ST486, DG75, CA46 and BL41 cells were transfected with lentivirus carrying shRNAs targeting circZDHHC11 or control vectors (NT1: black, NT2: grey, sh1: light red, sh2: dark red). Relative cell growth was assessed by following the percentage of GFP^+^ cells over three weeks post-transfection (n = 3), with the GFP percentage normalized to day 4 after transfection. Mean ± SD of three independent experiments is shown. Significance was determined by mixed model analysis; *p < 0.05, ***p < 0.001, ****p < 0.0001.

**Figure 3:**
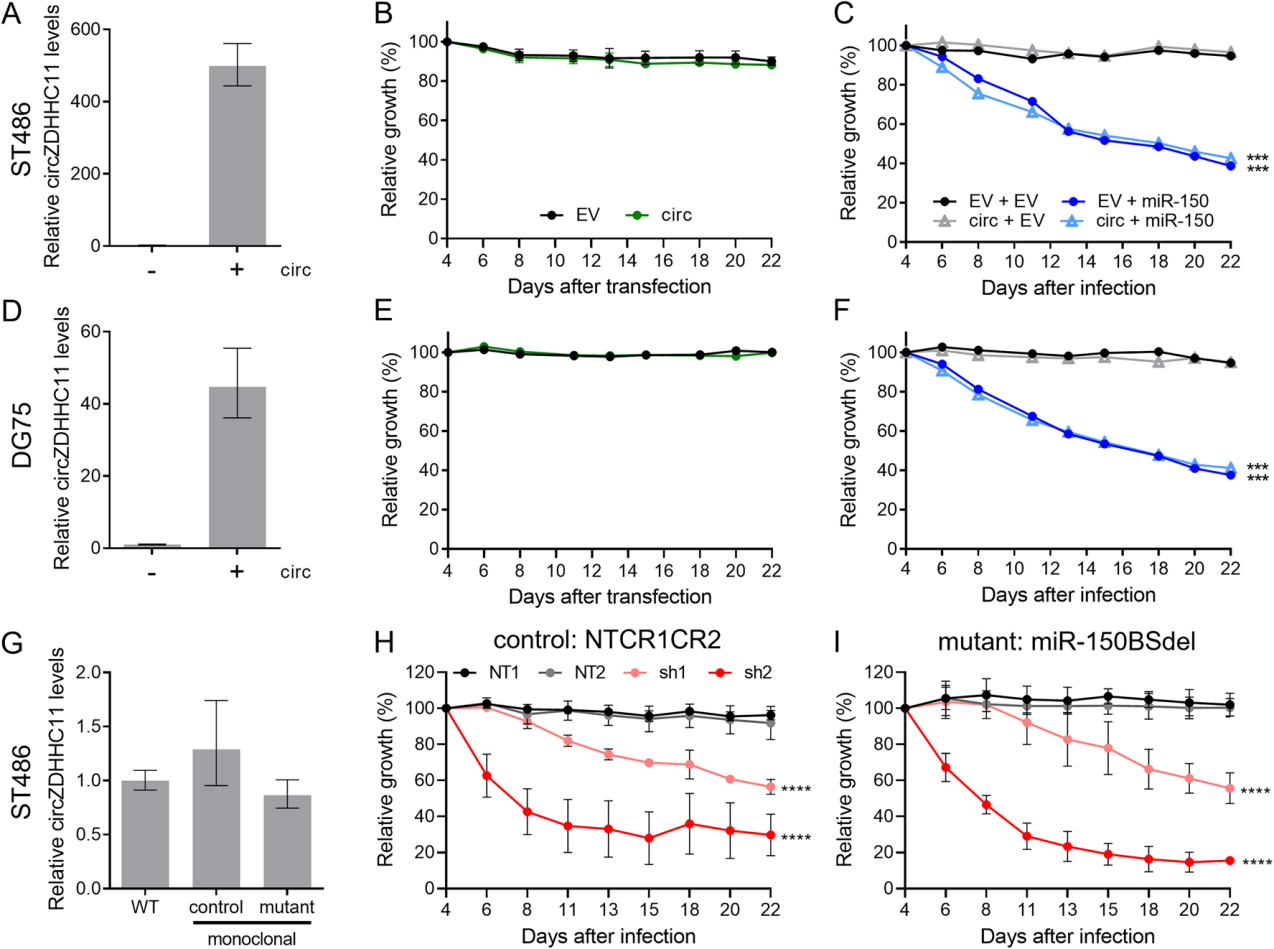
CircZDHHC11 overexpression does not rescue the effects of miR-150 overexpression and circZDHHC11 regulates growth independent of its miR-150 binding sites. (**A**) Effective overexpression of circZDHHC11 was obtained in ST486 upon infection with a lentiviral vector containing the circZDHH11 sequence. Mean expression of a representative experiment is shown with upper and lower limits. The levels in absence of circZDHHC11 overexpression are set to 1. (**B**) Relative cell growth of ST486 was assessed upon overexpression of circZDHHC11 (green) or infection with a control vector (black). The percentage of GFP^+^ cells was followed post-transfection for three weeks and the GFP percentage was normalized to day 4 after transfection. Mean ± SD of two independent experiments is shown. (**C**) ST486 cells transfected with the lentiviral vector for circZDHHC11 overexpression (triangles) or control vector (circles) (both with GFP reporter) were transfected with the lentiviral vector for miR-150 overexpression carrying a BFP reporter (dark and light blue) or control (black and grey). Relative cell growth was assessed by following the percentages of BFP^+^ cells in the GFP^+^ fraction over three weeks post-transfection, with the percentages normalized to day 4 after transfection. Significance was determined by mixed model analysis; ***p < 0.001. (D-F) Same as (**A**–**C**) in DG75 cells. (**G**) Expression levels of circZDHHC11 in WT ST486 and the monoclonal NTCR1CR2 control and miR-150BSdel mutant cell lines were analyzed by RT-qPCR. (**H**–**I**) The effect of circZDHHC11 knockdown on growth of the monoclonal NTCR1CR2 and miR-150BSdel cell lines cells (sh1: light red, sh2: dark red, NT1: black, NT2: grey). Relative cell growth was assessed by following the percentage of RFP^+^ cells over three weeks post-transfection from three independent experiments, with the RFP percentage normalized to day 4 after transfection. Significance was determined by mixed model analysis; ****p < 0.0001.

### Knockdown of circZDHHC11 inhibits Burkitt lymphoma cell growth.

To determine the relevance of circZDHHC11 for BL cell growth, we designed shRNAs which specifically knockdown circZDHHC11 without affecting the other ZDHHC11 transcripts. To achieve this, we designed two shRNAs targeting the back-splice junction of the circZDHHC11 transcript (Supplemental Fig. 1). Both shRNAs induced a clear knockdown of the circZDHHC11 transcript levels without affecting the levels of lncZDHHC11 and pcZDHHC11 transcripts (Supplemental Fig. 4 A,B). Knockdown of circZDHHC11 had a strong negative effect on growth in ST486 and more moderate effects in DG75, BL41 and CA46 (Fig. 2). In ST486, the decrease in the percentage of GFP^+^ cells was 55% for sh1 and 90% for sh2 at day 22. In DG75, both shRNAs resulted in a decrease of about 40% at day 22. In CA46 and BL41 a decrease in the percentage of GFP + of ~ 30% was observed for sh1 and no clear changes for sh2. These results showed that circZDHHC11 is important to maintain BL cell growth.

### Effect of circZDHHC11 knockdown on the network components and global gene expression.

In our previous work, we hypothesized that the ZDHHC11 transcripts ensure high levels of MYB by binding to miR-150, thereby stimulating growth of BL cells^1^. However, MYB protein and transcript levels were not changed upon circZDHHC11 knockdown, neither did we observe consistent changes for the other network components: MYC and miR-150 (Supplemental Fig. 4B,C,D). Thus, our results suggest that the effect of circZDHHC11 on BL growth does not involve MYB or other components of the network.

Next, we determined whether circZDHHC11 promotes BL growth by regulating the transcript levels of other genes by performing a genome-wide microarray gene expression analysis upon circZDHHC11 knockdown. To this end circZDHHC11 shRNA and control shRNA infected ST486 cells harvested on day 5 and 6 postinfection were analyzed. These timepoints were chosen as this was the timeframe with the most prominent drop in GFP + cells in the GFP competition assay (Fig. 2 and Supplemental Fig. 5A,B). Despite the strong effect on growth, we observed no significant changes in gene expression upon knockdown of circZDHHC11 (Supplemental Fig. 5C).

### circZDHHC11 overexpression does not rescue miR-150 induced inhibition of BL cell growth

We previously showed that miR-150 can strongly interact with the miR-150 BS region in circZDHHC11 and that circZDHHC11 was strongly increased in the AGO2-IP fraction upon miR-150 overexpression^1^. We therefore hypothesized that circZDHHC11 can rescue the miR-150 induced inhibition of BL cell growth. To test this, we overexpressed circZDHHC11 in two BL cell lines using a lentiviral overexpression vector. Effective overexpression of circZDHHC11 was confirmed by RT-qPCR with a fold induction of about 500 in ST486 and 45 in DG75, respectively (Fig. 3A,D). Sequence analysis of the exogenous circZDHHC11 including the BSJ region, confirmed proper formation of circZDHHC11 with a correct ligation of the 5’ to the 3’ ends (Supplemental Fig. 6A). The circular nature of the ectopically expressed circZDHHC11 was confirmed upon RNase R treatment (Supplemental Fig. 6B). Together, this indicated that the overexpressed circZDHHC11 has the correct sequence and the proper closed-loop structure. The exogenous circZDHHC11 showed a similar subcellular localization distribution as compared to the endogenous circZDHHC11 and its overexpression did not affect subcellular distribution of the other ZDHHC11 transcripts (Supplemental Fig. 6C). GFP competition assays showed no effect on growth of BL cells upon circZDHHC11 overexpression, suggesting that the endogenous circZDHHC11 levels are already sufficiently high to support growth of BL cells (Fig. 3B,E).

Next, we co-infected BL cells with the circZDHHC11 and the miR-150 overexpression vectors and analyzed potential effects on growth in a GFP competition assay. This revealed that the strong inhibition of cell growth upon miR-150 overexpression was not rescued by overexpression of circZDHHC11 (Fig. 3C,F). To confirm effective binding of miR-150 to exogenous circZDHHC11, we performed an AGO2 RNA immunoprecipitation experiment upon overexpression of circZDHHC11 in DG75 cells, in the absence or presence of miR-150 overexpression (Supplemental Fig. 6D). Overexpression of miR-150 resulted in enrichment of both miR-150 and circZDHHC11 in the AGO2-IP fraction, indicating that ectopically expressed circZDHHC11 does interact with miR-150. In line with the lack to rescue the phenotype upon miR-150 overexpression by circZDHHC11, we also observed a clear enrichment of MYB in the AGO2-IP fraction upon overexpression of miR-150, regardless of circZDHHC11 levels. This indicates that MYB is still targeted by miR-150 despite the high exogenous circZDHHC11 levels. Thus, although circZDHHC11 interacts with miR-150, overexpression of circZDHHC11 does not rescue the growth inhibiting effect of miR-150 overexpression.

### The miR-150 binding site region is dispensable for the growth supporting role of circZDHHC11

Since circZDHHC11 overexpression could not rescue the miR-150 induced phenotype, we next studied the importance of the miR-150 BS region for the growth supportive role of circZDHHC11. To this end, we removed the 488 bp miR-150 binding site region from the *ZDHHC11* locus in ST486 cells*.* A monoclonal cell line (miR-150BSdel) was generated using CRISPR/Cas9 technology with two guide RNAs flanking the miR-150 binding site region in ZDHHC11 (Supplemental Fig. 7A). Bi-allelic deletion of the miR-150 binding site region was confirmed by PCR on genomic DNA level and subsequent sequencing of the PCR product (Supplemental Fig. 7B). CircZDHHC11 expression levels in the miR-150BSdel monoclonal cell line were similar to the levels observed in unmodified cells and in a monoclonal cell line infected with 2 non-targeting sgRNAs (NTCR1CR2). This indicates that circZDHHC11 formation was not disturbed by deletion of the miR-150 binding site region (Fig. 3G). Knockdown of circZDHHC11 in the miR-150BSdel monoclonal cell line induced a clear negative effect on growth, with a decrease in the percentage of GFP^+^ cells that was comparable to the effect observed in the monoclonal control or WT cells (Fig. 3H–I and Fig. 2B). This indicated that the phenotype induced by knockdown of circZDHHC11 is independent of its ability to interact with miR-150 in the miR-150 binding site region.

## Discussion

It has been more than 30 years since the first circular RNAs were described in viroids^34^. Along with the development of high-throughput sequencing technology and computational pipelines, more and more circRNAs have been identified in different tissues and cell types. Genome-wide expression and functional studies have revealed novel insights with a broad spectrum of functionalities. However, the specific role and functional mechanism of the vast majority of circRNAs still needs to be determined. In recent years, more studies have focused on the role of circRNAs in different types of cancer. In this study, we showed that circZDHHC11 supports BL cell growth.

We previously postulated that the role of ZDHHC11 transcripts in the MYC/MYB/miR-150 oncogenic network was through sponging miR-150 and thereby preventing the miR-150-induced regulation of MYB. Based on the more prominent enrichment of circZDHHC11 in the AGO2-IP fraction upon miR-150 overexpression in BL as compared to the linear ZDHHHC11 transcripts, we hypothesized that especially the circZDHHC11 transcript would be critical to achieve effective sponging of miR-150^1^. In line with this hypothesis, we here showed a cytoplasmic localization for both miR-150 and circZDHHC11. The predominant nuclear localization of pcZDHHC11 and lncZDHHC11 argued against a strong interaction of these transcripts with miR-150. Previously, we showed that knockdown of all ZDHHC11 transcripts simultaneously reduced growth of BL cells. The ZDHHC11 knockdown induced a reduction in MYB levels, which might have been caused by a more effective inhibition of MYB due to the release of miR-150 from ZDHHC11 transcripts^1^. We now showed that circZDHHC11 supports BL cell growth by selective knockdown of this transcript, without affecting the linear ZDHHC11 transcripts. In contrast to the effect of knockdown of all three types of ZDHHC11 transcripts simultaneously, knockdown of circZDHHC11 did not decrease MYB levels. This might in part be explained by the much lower endogenous levels of circZDHHC11 as compared to the two linear ZDHHC11 transcripts. Although the higher overall levels of the linear transcripts potentially make them more efficient miR-150 sponges, the subcellular localization experiments do not support this function.

To further understand the growth supporting role of circZDHHC11, we determined whether it could influence gene expression. No effects were observed on genes within the network and neither genome-wide. Although our results are consistent with the predominant cytoplasmic localization of circZDHHC11, it is quite unexpected that no (secondary) effects at the transcript level were observed in cells showing a very clear growth-related phenotype. Our current data suggest that circZDHHC11 more likely acts at the posttranscriptional level. However, we cannot exclude that minor changes on gene expression are missed.

Ectopic expression of circZDHHC11 did not have an effect on BL cell growth, suggesting that the relatively low endogenous circZDHHC11 levels are sufficient to support growth. It would be interesting to explore a potential growth supporting effect of circZDHHC11 overexpression under suboptimal growth conditions, e.g. low serum, low oxygen or low cell density. Remarkably, overexpression of circZDHHC11 in BL cells overexpressing miR-150 did not result in any rescue of the growth inhibitory phenotype upon miR-150 overexpression. This was unexpected since we did confirm a cytoplasmic localization similar to the endogenous circZDHHC11 and confirmed its ability to interact with miR-150 using AGO2-IP. These experiments indicate that circZDHHC11 supports growth independent of its interaction with miR-150.

In line with these results, we showed that knockdown of circZDHHC11 in cells lacking the miR-150 binding site region, still inhibited growth of BL cells. Moreover, being able to generate a monoclonal cell line with this deletion argues against an important role of the miR-150 binding site region in circZDHHC11 and possibly any of the other ZDHHC11 transcripts in supporting growth of BL cells. The ZDHHC11 protein or the ZDHHC11 lncRNA might influence MYB levels via palmitoylation or other direct or indirect mechanisms. Our current results point towards a function of circZDHHC11 which is independent of sequestering miR-150, potentially by binding to and modifying functionality of other molecules.

To further elucidate the role of circZDHHC11, pulldown experiments need to be set up to identify its binding partners. In addition, deleting or mutating additional regions of circZDHHC11 will allow pinpointing the most critical region(s) and aid in defining the most critical interactor(s). Functions beyond acting as a sponge or as a transcriptional regulator have been described for other circRNAs with strong miRNA binding domains. For circRNA CDR1as, which is a well-known miR-7 sponge, it was shown that it can stabilize p53 protein independent of its sponge function in glioma^35^ and silencing of CDR1as drives IGF2BP3-mediated progression independent of miR-7 in melanoma^36^. Finally, once interactors have been identified their relevance should be studied in relation to the observed phenotype upon circZDHHC11 knockdown. We previously already showed that circZDHHC11 can be detected in primary BL cases albeit at variable levels^1^. Expression of the potential interactors should also be determined in primary BL cases.

In conclusion, we showed that circZDHHC11 is critical for BL cell growth independent of the miR-150 binding site region. The effect on BL cell growth is most likely at the post-transcriptional level, potentially via interaction with other cytoplasmic RNA transcripts or proteins.

### Supplementary Information

Supplementary Information.

## Data Availability

All data generated or analyzed during this study are included in this published article [and its supplementary information files].
