# Supplementary material for: Circular ZDHHC11 supports Burkitt lymphoma growth independent of its miR-150 binding capacity
Source: Sci Rep. 2024 Apr 16;14:8730. doi: 10.1038/s41598-024-59443-3 (PMC11021472; doi:10.1038/s41598-024-59443-3)
Supplement: Supplementary file 1 — Supplementary Information. [file 41598_2024_59443_MOESM1_ESM.pdf]

Circular ZDHHC11 supports Burkitt lymphoma growth independent of its miR-150 binding capacity

Yichen Liu[1,2], Xing Zhao[1], Annika Seitz[1], Annie A. Hooijsma[1], Reyhaneh Ravanbakhsh[1,3], Sofia Sheveleva[1], Debora de Jong[1], Jasper Koerts[1], Agnieszka Dzikiewicz-Krawczyk[4] , Anke van den Berg[1], Lotteke J.Y.M Ziel-Swier[1*], Joost Kluiver[1*]

[1]Department of Pathology and Medical Biology, University of Groningen, University Medical Center Groningen, Groningen, the Netherlands. [2]Cancer Hospital Academy of Medical Sciences and Peking Union Medical College, China. [3]Department of Aquatic Biotechnology, Artemia and Aquaculture Research Institute, Urmia University, Urmia, Iran. [4]Institute of Human Genetics, Polish Academy of Sciences, Poznan, Poland

[*]authors share the last authorship

Corresponding authors: Dr. Joost Kluiver, Department of Pathology and Medical Biology, University Medical Center Groningen, Hanzeplein 1, 9700 RB Groningen, The Netherlands. Tel: +31503618075; Fax: +31503619107; Email: j.l.kluiver@umcg.nl

Conflict of Interest Statement: The authors declare no conflicts of interest.

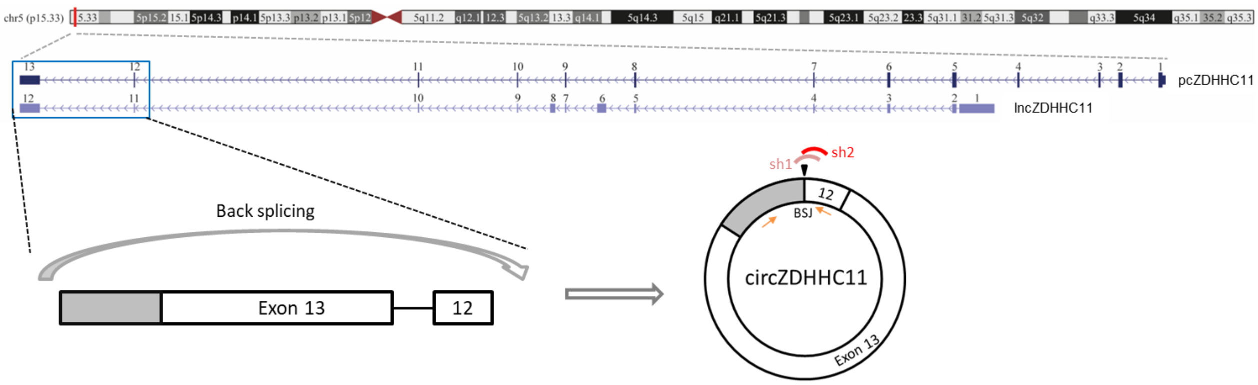

Supplemental Figure 1: ZDHHC11 locus and encoded transcripts. Schematic representation of the *ZDHHC11* locus and the three ZDHHC11 transcripts: protein-coding (pcZDHHC11), lncRNA (lncZDHHC11) and circRNA (circZDHHC11). The circZDHHC11 transcript is formed by back splicing of exon 12, exon 13 and a part of sequence following exon 13. The black triangle indicates the back-splice junction (BSJ) of circZDHHC11. The circZDHHC11 specific shRNAs (sh1 and sh2) targeting the BSJ region are indicated with curved lines, and the RT-qPCR primer set is indicated with the orange arrows.

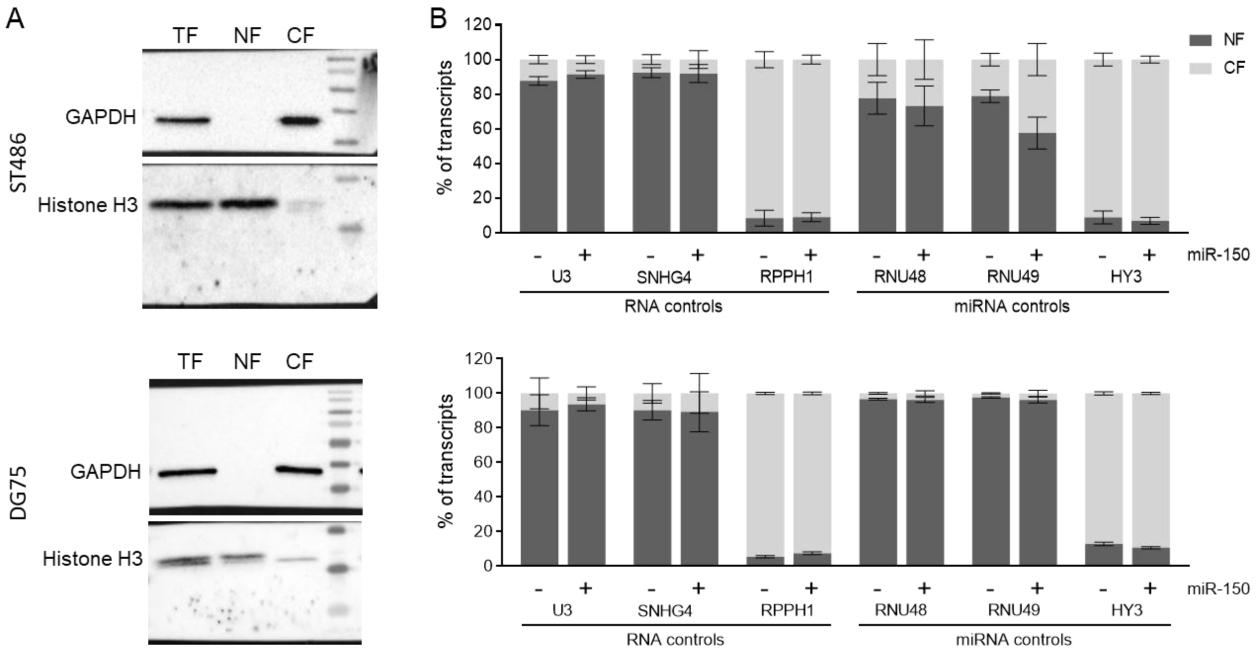

Supplemental Figure 2: Control experiments to determine the efficiency of the cell fractionation for ST486 and DG75 cells. (A) Western blots showing the presence of GAPDH in the cytoplasmic fraction (CF) and histone H3 in the nuclear fraction (NF) of ST486 and DG75 cells. Both proteins are also detected in the total fraction (TF). The original uncropped gel images can be found in supplemental figure 4. (B) Subcellular localization validation qPCR of control RNA transcripts with known subcellar localization, in ST486 and DG75 cells in the absence (-) or presence (+) of miR-150 overexpression. U3, SNHG4, RNU48 and RNU49 are located in the nucleus, while RPPH1 and HY3 are located in the cytoplasm. The percentage of transcripts located in the cytoplasm (light grey, CF) and nucleus (dark grey, NF) are shown as mean ± SD of two independent experiments.

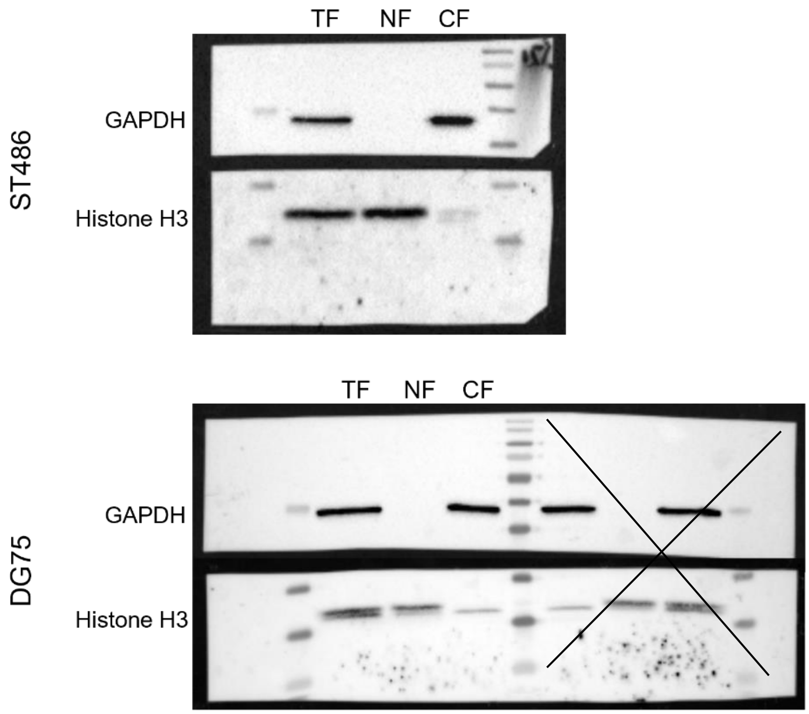

Supplemental Figure 3: Original pictures of the blots of the control experiments to determine the efficiency of the cell fractionation for ST486 and DG75 cells. Original uncropped images of the western blots shown in Supplemental figure 2A.

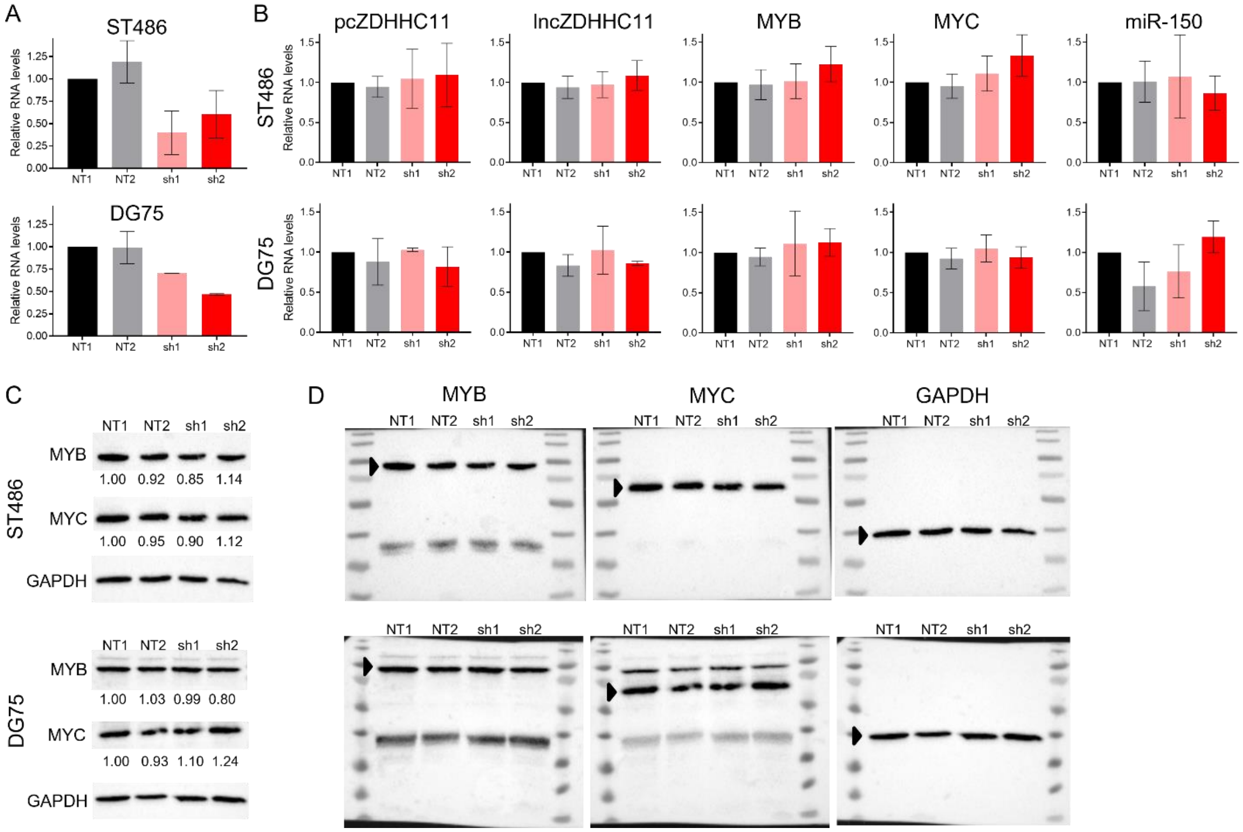

Supplemental Figure 4: circZDHHC11 knockdown efficiency and its effects on the expression of linear *ZDHHC11* transcripts and other network components. (A) Analysis of the circZDHHC11 KD efficiency. The expression of circZDHHC11 was measured by RT-qPCR in ST486 and DG75 cells transfected with lentiviral vectors carrying shRNA (sh1: light red, sh2: dark red) or control (NT1: black, NT2: grey) sequences. Error bars indicate mean ± SD of three (ST486) or two (DG75) independent experiments, and the data were normalized to the NT1 control. (B) Effect of circZDHHC11 knockdown on expression of pcZDHHC11, lncZDHHC11 and other network components in ST486 and DG75. (C) Western blots showing the effect of circZDHHC11 knockdown on MYB and MYC protein levels. Quantification of MYB and MYC protein levels are normalized to GAPDH. The levels of NT1 were set to 1 for comparison between conditions and cell lines. (C) Original pictures of the blots shown in (D). The triangles indicate the position of the specific bands of MYB, MYC and GAPDH. The blots were incubated in the multiple antibody

solutions (first with antibody against MYB, secondly with antibody against MYC and finaly with antibody against GAPDH), with stripping of the blots performed in between the incubations.

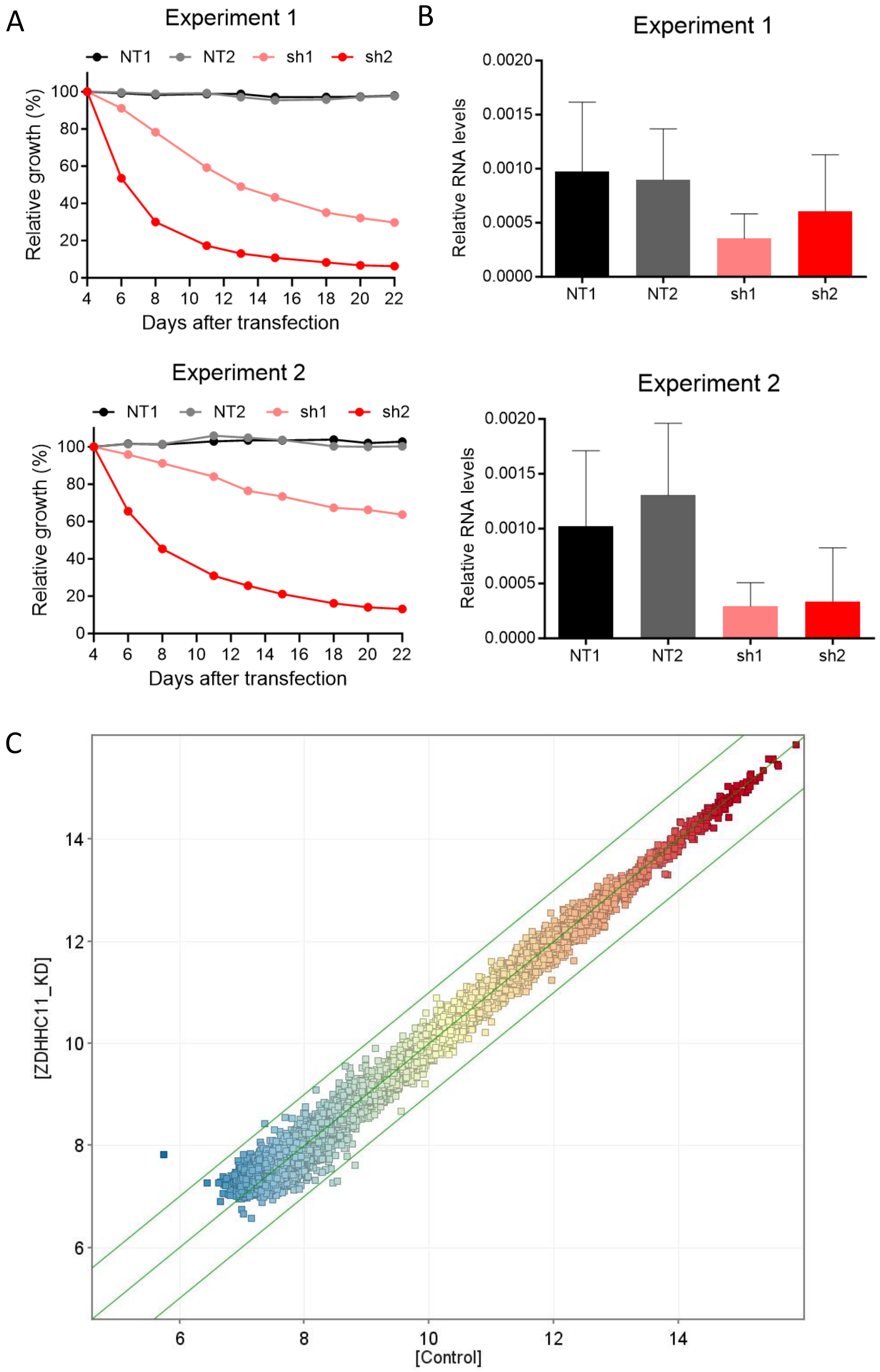
A
Experiment 1
NT1
NT2
sh1
sh2
Relative growth (%)
0
20
40
60
80
100
4
6
8
10
12
14
16
18
20
22
Days after transfection
Experiment 2
NT1
NT2
sh1
sh2
Relative growth (%)
Days after transfection
B
Experiment 1
Relative RNA levels
0.0000
0.0005
0.0010
0.0015
0.0020
NT1
NT2
sh1
sh2
Experiment 2
Relative RNA levels
NT1
NT2
sh1
sh2
C
[ZDHHC11_KD]
[Control]
6
8
10
12
14

Supplemental Figure 5: CircZDHHC11 KD does not induce significant changes in gene expression. (A) GFP assays to confirm the expected phenotype upon circZDHHC11 knockdown for two independent infections that were done to generate samples for microarray analysis. (B) RT-qPCR analysis to confirm efficient knockdown of circZDHHC11. Analysis was performed on the same RNA as used for microarray analysis. RNA was isolated from GFP-sorted cells on day 5 (experiment 1) or day 6 (experiment 2). (C) Scatterplot comparing circZDHHC11 shRNAs (sh1 and sh2 combined, y-axis) vs control shRNA (NT1, x-axis). No significant changes in gene expression were observed. Outer green lines indicate 2-fold change, middle green line indicates the no change line.

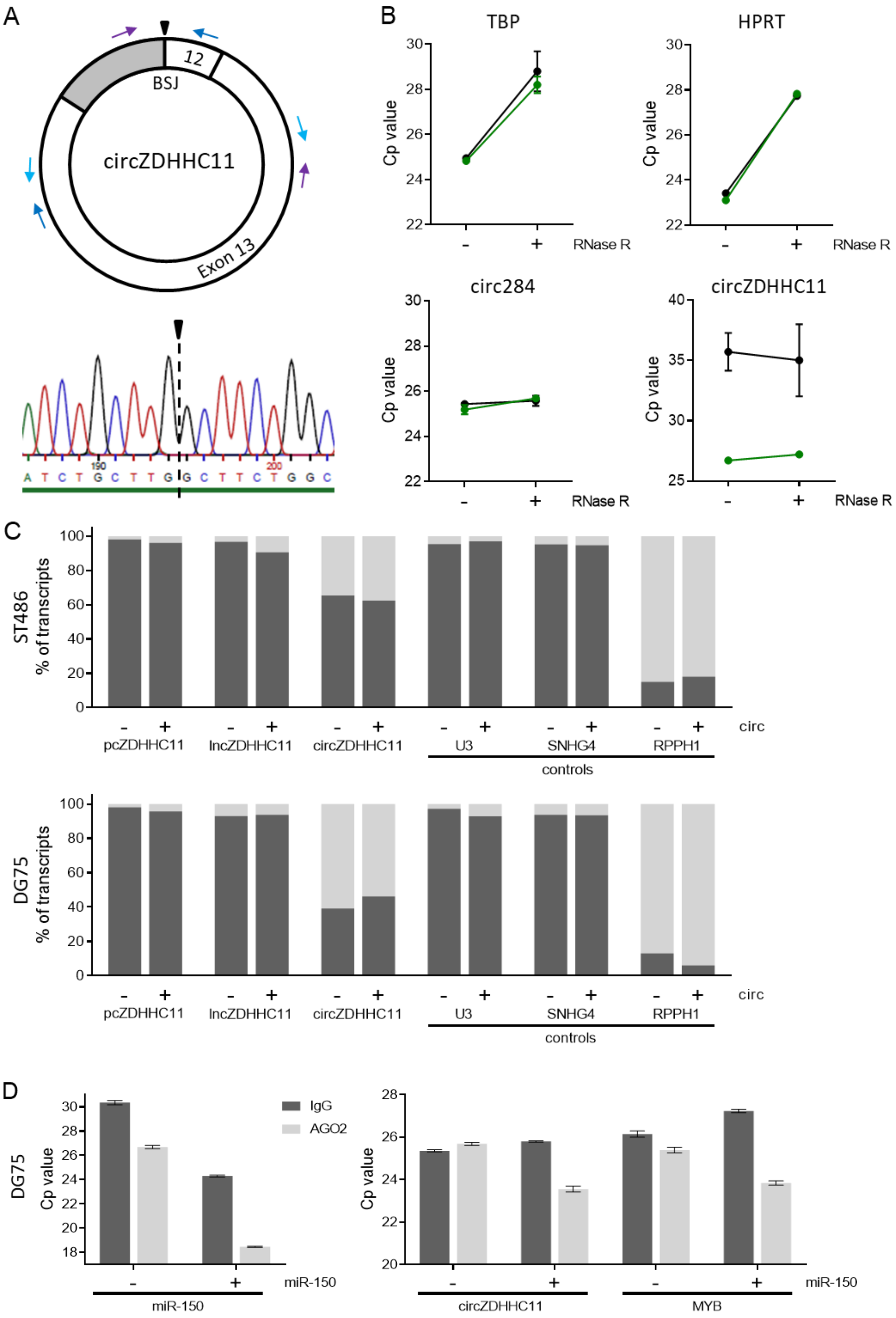
A
12
BSJ
circZDHHC11
Exon 13
A T C T G C T T G G C T T C T G G C
B
TBP
HPRT
circ284
circZDHHC11
Cp value
RNase R
C
ST486
% of transcripts
DG75
% of transcripts
circ
pcZDHHC11
lncZDHHC11
circZDHHC11
U3
SNHG4
RPPH1
controls
D
DG75
Cp value
IgG
AGO2
miR-150
circZDHHC11
MYB

Supplemental Figure 6: Validating overexpression of circZDHHC11. (A) Schematic representation of the circZDHHC11 structure with the arrows indicating the primers used for sequencing of the circZDHHC11 sequence. Sequence results covering the BSJ showed the expected sequence. The BSJ is indicated by the black triangle. (B) Confirmation of the circular nature of the exogenous circZDHHC11 product by RNase R treatment of the RNA samples before RT-qPCR (circZDHHC11 overexpression: green, control: black; - and + indicate the absence or presence of RNase R). Effective RNase R treatment was shown for controls TBP and HPRT. Hsa_circ_0000284 was taken as a positive control for circRNAs being protected from RNase R degradation. Mean ± SD of three experiments are shown. (C) Subcellular localization of the *ZDHHC11* transcripts and control RNA transcripts with known subcellular localization in the absence (-) or presence (+) of circZDHHC11 overexpression in ST486 and DG75 cells. The fraction of the total RNA present in the cytoplasm (light grey) and nucleus (dark grey) is indicated. U3 and SNHG4 are located in the nucleus, while RPPH1 is located in the cytoplasm. (D) qPCR of the AGO2 (light grey) and IgG (dark grey) immunoprecipitation fractions showing the Cp values for miR-150, circZDHHC11 and MYB upon overexpression of circZDHHC11 and in the absence (-) or presence (+) of miR-150 overexpression.

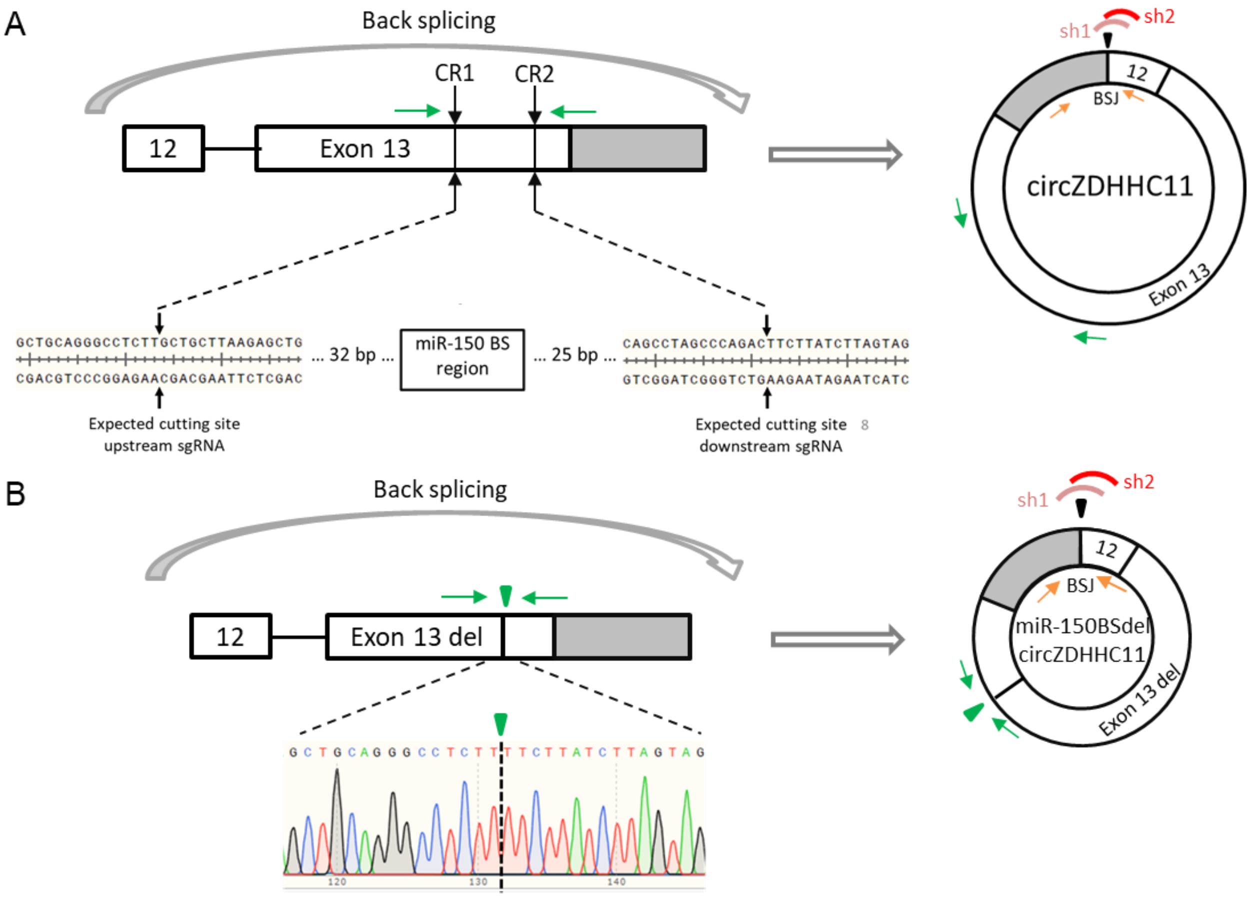

Supplemental Figure 7: Generation of the miR-150BSdel monoclonal cell line. (A) ST486 cells were infected with a lentiviral CRISPR/Cas9 vector containing two single guide RNAs (sgRNA CR1 and CR2, indicated by the two black arrows), which flank the miR-150 binding site (BS) region in ZDHHC11. The expected cutting sites of the sgRNAs in the *ZDHHC11* sequence are indicated with two black arrow sets, with in between the miR-150 BS region. The black triangle indicates the BSJ of circZDHHC11, the red bend lines the circZDHHC11 specific shRNAs and the orange arrow set indicates the positions of the circZDHHC11 specific qPCR primers. (B) Sanger sequencing showed the deletion of the miR-150 BS region in the *ZDHHC11* locus of the monoclonal miR-150 BS del cell line. Primers for sequencing are indicated with the green arrow set. The green triangle indicates the junction point after deletion of the miR-150 BS region.

Supplemental Table 1: Oligonucleotide sequences.

Name	Sequence (5'-3')
qPCR primers	
circZDHHC11-F	GGCATCCTCTGTATTTTAATGAACTCT
circZDHHC11-R	GTTGCAGCCAGAAGCCAA
pcZDHHC11-F	TGTCGAGCACTCCCCAGA
pcZDHHC11-R	GCAACGGAAAACGGCTCTC
lncZDHHC11-F	AGCACTTCCTGAAAGCCAGC
lncZDHHC11-R	GAAGAACGCAACAGGCATCG
MYC-F	GCTCATTTCTGAAGAGGACTTGTTG
MYC-R	TTACGCACAAGAGTTCCGTAGCT
MYB-F	CCAACTGTTCACGCAGACCT
MYB-R	CTTCTGATGCTGGTGCCATT
U3-F	AACCCCGAGGAAGAGAGGTA
U3-R	CACTCCCCAATACGGAGAGA
SNHG4-F	AGTAGGGCATCCTTCACCCA
SNHG4-R	CCCTACCCCCATCTGAGCTAT
RPPH1-F	AGCTTGGAACAGACTCACGG
RPPH1-R	AATGGGCGGAGGAGAGTAGT
TBP-F	GCCCGAAACGCCGAATAT
TBP-R	CCGTGGTTCGTGGCTCTCT
HPRT-F	GGCAGTATAATCCAAAGATGGTCAA
HPRT-R	GTCTGGCTTATATCCAACACTTCGT
circ284-F	ATTATGTTGGTGGATCCTGTT
circ284-R	ATATGGTGGGTAGACCAAGA
PCR primers	
circZDHHC11seq-F1	CTTGGCTTCTGGCTGCAAC
circZDHHC11seq-R1	AGCACAGTTAAGCAGGTGGC
circZDHHC11seq-F2	TCTGAGTTTTTCAGCCTAGCCC
circZDHHC11seq-R2	GGGCTCTGTTGTTTCTTGTTGC
circZDHHC11seq-F3	CCAGCATCCATCTCTGTCATCATC
circZDHHC11seq-R3	AATGACTACTAAACAGATGAAAAT
sgCR1CR2-F	CATCCATCTCTGTCATCATC
sgCR1CR2-R	AATGACTACTAAACAGATGAAAAT
sgRNAs	
sgCR1	ATCAGCTCTTAAGCAGCAAG
sgCR2	GTCTACTAAGATAAGAAGTC
sgNTCR1	ACGGAGGCTAAGCGTCGCAA
sgNTCR2	ATCGTTTCCGCTTAACGGCG
shRNAs	
sh1 S	GATCCGCTTGGCTTCTGGCTGCAACTTCAAGAGAGTTGCAGCCAGAAGCCAAGCTTTTTG
sh1 AS	AATTCAAAAAGCTTGGCTTCTGGCTGCAACTCTCTTGAAGTTGCAGCCAGAAGCCAAGCG
sh2 S	GATCCGGCTTCTGGCTGCAACAAGAATTCAAGAGATTCTTGTTGCAGCCAGAAGCCTTTTTG
sh2 AS	AATTCAAAAAGGCTTCTGGCTGCAACAAGAATCTCTTGAATTCTTGTTGCAGCCAGAAGCCG

Supplemental Table 2: Overview of antibodies used for Western blotting.

Name	Dilution	Catalog number, company
rabbit monoclonal anti-c-MYC [Y69]	1:1000	ab32072, Abcam, Cambridge, UK
rat monoclonal anti-c-Myb [ANA236B] (C-terminal)	1:500	ab169111, Abcam, Cambridge, UK
mouse monoclonal anti-GAPDH antibody	1:50000	NB600-502, Novus Biologicals, UK
rabbit polyclonal histone H3 antibody (FL-136)	1:500	sc-10809, Santa Cruz Biotechnology, USA
goat anti-Rabbit immunoglobulins/HRP	1:1000	P0448, Dako, USA
goat anti-mouse immunoglobulins/HRP	1:1000	P0447, Dako, USA
rabbit anti-goat immunoglobulins/HRP	1:1000	P0449, Dako, USA
